# Editorial: Fertility preservation in female cancer survivors: innovative strategies for fertility preservation and reproductive outcomes

**DOI:** 10.3389/fendo.2026.1999787

**Published:** 2026-09-29

**Authors:** Giacomo Corrado, Lucrezia Massaro, Stefano Canosa, Erica Silvestris

**Affiliations:** 1 Dipartimento Scienze della Salute della Donna e del Bambino, UOC Ginecologia Oncologica, Fondazione Policlinico Universitario A. Gemelli IRCCS, Rome, Italy; 2 Centre for Research and Studies on Procreative Health, University Department of Life Sciences and Public Health, Università Cattolica del Sacro Cuore, Rome, Italy; 3 Obstetrics and Gynecology 1U, Physiopathology of Reproduction and IVF Unit, Department of Surgical Sciences, Sant Anna Hospital, University of Turin, Turin, Italy; 4 Gynecologic Oncology Unit, IRCCS Istituto Tumori ‘Giovanni Paolo II’, Bari, Italy

**Keywords:** cancer survivorship, counselling, fertility preservation, oncofertility, reproductive intention

Advances in cancer diagnosis and treatment have markedly improved survival, shifting the goals of care from survival alone toward long-term quality of life and survivorship. In this context, preserving the reproductive potential of girls and women diagnosed with cancer before or during their reproductive years has become an increasingly important component of comprehensive oncological care. The gonadotoxic effects of chemotherapy, radiotherapy, and some surgical treatments are now well recognised, making fertility preservation (FP) a relevant consideration from the time of diagnosis. The contributions to this Research Topic address this complex field from complementary perspectives, spanning patients’ knowledge and concerns, communication and decision support, established and emerging FP strategies, disease-specific challenges, and reproductive outcomes.

The psychosocial dimension of oncofertility emerges as a central theme. A cross-sectional survey of women referred for specialist counselling showed high awareness of treatment-related infertility and substantial interest in FP, while also identifying persistent informational gaps and concerns about treatment delay, safety, effectiveness, and cost (Corrado et al.). A systematic review and meta-analysis further demonstrated that fertility concerns are shaped by multiple individual and clinical factors: higher education, fertility intentions, endocrine therapy, and previous reproductive counselling were associated with greater concerns, whereas having more than one child and being married were associated with lower levels of concern (Wang et al.). These apparently paradoxical findings underline that information alone is not sufficient; counselling must be timely, individualised, and responsive to patients’ psychological state, reproductive goals, and social context. Complementing the patient perspective, a qualitative evidence synthesis of healthcare professionals identified organisational culture, geographic and policy barriers, limited FP knowledge, weak interprofessional connections, and suboptimal communication as important obstacles to effective decision support (Jiang et al.).

Appropriate counselling should therefore provide a clear overview of available strategies while considering age, pubertal status, cancer type, treatment urgency, and expected gonadotoxicity. Established options include oocyte and embryo cryopreservation, ovarian tissue cryopreservation (OTC), and, in selected settings, ovarian transposition and fertility-sparing surgery (Yazdani et al). Oocyte and embryo cryopreservation generally require controlled ovarian stimulation and are therefore applicable to post pubertal patients, whereas OTC can be offered to prepubertal girls and to patients who cannot postpone anticancer treatment. Surgical approaches, including ovarian transposition before pelvic radiotherapy, further broaden the possibilities for carefully selected patients (Yazdani et al).

Disease-specific considerations remain crucial. Leukemia and other haematological malignancies pose particular challenges because of treatment urgency and the potential presence of malignant cells in ovarian tissue, which may make subsequent tissue reimplantation unsafe (Abazarikia et al., Salvagno et al.). These concerns are especially relevant in paediatric patients, for whom OTC may represent the only established FP option (Salvagno et al.). Emerging strategies including artificial ovary construction, *in vitro* follicle growth, stem-cell-based neo-folliculogenesis, and ex vivo purging of malignant cells aim to preserve fertility while reducing the risk of disease reintroduction, although they remain largely experimental (Abazarikia et al., Salvagno et al.).

Other clinical scenarios also require highly individualised management. In pregnancy-associated cancer, FP must be coordinated with obstetric and oncological treatment, and current guidelines still provide limited specific recommendations for this population (Nilsson et al.). In early breast cancer, available evidence supports the feasibility of pregnancy after treatment, including in selected patients with hormone receptor-positive disease, without evidence of worsened oncological outcomes in the populations studied (Turla et al.). For early cervical cancer, long-term data support radical trachelectomy as an effective fertility-sparing option in appropriately selected patients, with favourable oncological and reproductive outcomes (Liu et al.).

Several contributions also explore strategies to improve the efficacy of current FP techniques. In a xenograft model, human ovarian cortical tissue showed more efficient revascularisation and less apoptosis when the medullary interface faced the vascular bed, suggesting that graft orientation may influence early tissue survival after transplantation (Cheng et al.). In another preclinical study, α-lipoic acid reduced oxidative damage and follicular abnormalities in vitrified cat ovarian tissue, supporting further investigation of antioxidant strategies to optimise cryopreservation (Yin et al.).

In women of advanced maternal age undergoing IVF-ET, the ovarian sensitivity index (OSI) was associated with ovarian response and intermediate reproductive outcomes but not with live birth, suggesting a role in tailoring ovarian stimulation rather than predicting ultimate reproductive success (Zhong et al.).

Taken together, these studies reinforce the view that oncofertility counselling should not be considered an ancillary service, but an integral component of patient-centred cancer care. Important gaps nevertheless remain, particularly in access to specialised pathways, multidisciplinary coordination, ethical and legal regulation, and the long-term management and disposition of cryopreserved reproductive material (Wang et al.). Building structured oncofertility networks with clear referral pathways, dedicated multidisciplinary teams, and longitudinal follow-up could reduce delays, improve continuity of care, and support more equitable access. Ultimately, fertility preservation requires an approach that integrates oncological safety with reproductive potential while also recognising the emotional, psychological, social, relational, and ethical dimensions of cancer survivorship.

